# Recommendations for designing genetic test reports to be understood by patients and non-specialists

**DOI:** 10.1038/s41431-020-0579-y

**Published:** 2020-02-05

**Authors:** George D. Farmer, Harry Gray, Gemma Chandratillake, F Lucy Raymond, Alexandra L. J. Freeman

**Affiliations:** 1 Winton Centre for Risk & Evidence Communication, University of Cambridge, Cambridge, CB3 0WA UK; 2 Division of Neuroscience & Experimental Psychology, University of Manchester, Manchester, M13 9PL UK; 3 Leverhulme Research Centre for Forensic Science, University of Dundee, Dundee, DD1 4HN UK; 4 MRC Biostatistics Unit, University of Cambridge, Cambridge, CB2 0SR UK; 5 East Midlands & East of England NHS Genomic Medicine Service, Cambridge, CB2 0QQ UK; 6 Institute of Continuing Education, University of Cambridge, Cambridge, CB23 8AQ UK; 7 Department of Medical Genetics, Cambridge Institute for Medical Research, Cambridge, CB2 0XY UK

**Keywords:** Patient education, Human behaviour

## Abstract

Patients and non-specialist healthcare professionals are increasingly expected to understand and interpret the results of genetic or genomic testing. These results are currently reported using a variety of templates, containing different amounts, levels, and layouts of information. We set out to establish a set of recommendations for communicating genetic test results to non-expert readers. We employed a qualitative-descriptive study design with user-centred design principles, including a mixture of in-person semi-structured interviews and online questionnaires with patients, healthcare professionals and the general public. The resulting recommendations and example template include providing at-a-glance comprehension of what the test results mean for the patient; suggested next steps; and details of further information and support. Separation and inclusion of technical methodological details enhances non-specialists’ understanding, while retaining important information for specialists and the patients’ records. The recommendations address the high-level needs of patients and their non-specialist clinicians when receiving genetic test results. These recommendations provide a solid foundation for the major content and structure of reports, and we recommend further engagement with patients and clinicians to tailor reports to specific types of test and results.

## Introduction

Genetic and genomic testing is predicted to have a greatly increased role in healthcare [1]. In practice this means that such testing is increasingly likely to be ordered, and the results interpreted, by non-specialist clinicians involved in routine patient care. Under the broader directives of shared decision-making and less paternalistic medicine, patients themselves also have an increasing desire to receive and understand detailed information about their unique genetic make-up and its consequences for their lives. Genetic test reports, therefore, need to be able to deliver the results and their implications clearly and unambiguously to those who have no training in genetics.

This is no small challenge. Even in the simple case of genetic testing in clinical healthcare there are multiple domains of uncertainty surrounding the result itself (e.g. testing error) and what it actually means (e.g. variant significance). These challenges are exacerbated as more genomic regions are tested, since a greater quantity of errors will be induced (even for a low testing error-rate) and more variants will be of ‘uncertain significance’ as more clinically uncertain loci of the genome are considered.

This point has been recognised for some time now by leading national governing bodies for genetics. As a result, guidelines for genetic test reporting have been released by the American College of Medical Genetics and Genomics (ACMG) [2], European Society of Human Genetics (ESHG) [3], and Association for Clinical Genetic Science (ACGS) [4]. These guidelines are primarily concerned with reporting the technical details associated with the testing procedure and result interpretation. Importantly, they all vary in their recommendations [5] and interpretation (see O’Daniel et al. [6] for a US example) and are perpetually revised as testing methodologies and the clinical relevance of genetic variations become better understood. This results in the need for extremely clear communication of exactly what was tested and what the result means in every single report so that non-specialists (and specialists alike) can readily interpret reports without requiring contextual knowledge of the reporting landscape at the time of testing and from the location in which the report was generated. The requirement for clear communication (including to non-experts) is stated in the guidelines for all of the organisations listed above but isn’t explicitly elaborated on any further. In fact, the practical guidance for ensuring this clear communication of results is acknowledged to be minimal.

## Review of existing knowledge

Efforts have been made to assess the communication of results in reports from a variety of testing contexts for both clinicians and patients, leading to the development of communication guidelines and suggested report formats (e.g. Lubin et al. [7] and Haga et al. [8]).

There are a few common themes. First, authors have commented on the relevance of information contained within reports. Joseph et al. [9] noted the discrepancies between the information that genetic counsellors provided to patients versus the information that the patients indicated caring most about. Given that it is experts in genetics who have traditionally decided the content and format of reports, we identified this as likely to be an ongoing issue to be addressed.

Stuckey et al. [10] found that parents in their study continually searched for relevant information and resources for their child’s genetic condition, but that current reporting didn’t meet that need and led parents to extensive online searching, with some admitting reticence relating to the information that they found, and at least one saying that she scared herself. Not all patients want more information, though: Joseph et al. [9] described how one patient didn’t want to know about information regarding the test, genetics, or risk since they were factors beyond their control—only the outcome was seen as meaningful. Williams et al. [11] also looked at reporting from a patient perspective, identifying that with the complex results of whole-genome sequencing, some supportive interpretation information was necessary to communicate potential clinical relevance to their patient. They also appreciated that active clinical guidance was given so that they could confidently convey this to the patient at the time of reading. The challenge for reports is to provide information that is seen as relevant for all parties, while being concise enough to not deter patients.

A second issue is the communication of the uncertainties inherent in genetic tests and their results. These can be classified into two kinds: genetic risk (the estimated chance that a person will experience a given consequence) and testing limitations (the chance that estimate is inaccurate). Perceptions of genetic risk information have been shown to be affected by individuals’ preconceptions of testing [12], which can have its roots in familial and cultural backgrounds [8]. While managing testing expectations is the role of the genetic counsellor, there is considerable evidence in the literature concerning risk communication strategies that could be used to build a robust report template. A comprehensive review on this topic is provided in Lautenbach et al. [13], which pools together the literature for genetic risk communication with that of general medical risk. It highlights important messages that are echoed throughout the risk communication world: there is no one-size-fits-all approach and so multiple methods of communication should be presented, positive and negative framing of risk should both be given in order to avoid framing bias, and pictographs can be used as effective methods of communicating percentages. Shaer et al. [14] empirically demonstrated that visual presentations of genetic variant information (regarding pathogenicity and clinical importance) to non-experts showed improved comprehension and perceived comprehension when compared with traditional tabular design formats. Adding the uncertainty resulting from limitations to the testing accuracy compounds this complex landscape of risk information but is necessary. Both Dorschner et al. [15] and Kelman et al. [16] have reported clinicians as wanting access to false positive and false negative rates of tests for themselves and patients, although it is uncertain from the literature whether patients would find this kind of information understandable and useful, and it is seldom mentioned in current reports.

A third theme is the need for citations of scientific evidence from which conclusions in the report derive, and available resources for further information. In focus groups, Cutting et al. [17] found clinicians wanted citations to the academic literature that supports the interpretations stated in the report. Johnson et al. [18], taking a more patient-centric approach, found interviewees also wished to see additional information about genomic testing, as well as the actions of others in similar situations.

Finally, unsurprisingly, the language used throughout the report has been seen as critical to users’ understanding. Studies have shown that non-experts prefer simpler language [9, 11], with technical genetic terms creating confusion and reluctance to even try to understand the content [10]. Aside from this negative perception of technical language, it has also been shown that there is a positive perception of ‘simpler’ language. Lewis et al. [19] showed that simpler language facilitated understanding and confidence to talk about results.

One theme with relatively little available evidence in the literature is the graphic design and layout of reports. This is interesting because there is a stark contrast between the visual appearance of reports that have been developed by, for example, direct-to-consumer (DTC) genetic testing companies compared with those suggested in the academic literature; the former tend to make more use of colour and graphical elements to separate sections of their reports when compared with the latter. It is likely that these private companies have conducted testing with their users that shows preference for graphics, but that that data is proprietary. More data needs to be gathered on this topic in the public domain when shifting focus towards patients as recipients, since it may directly increase comprehension [14].

In light of the limitations identified in this review of the literature, and the lack of specific official guidance, we undertook qualitative research with an objective of providing recommendations for making the content and structure of a genetic test report more accessible to patients and non-specialist clinicians. To achieve this we undertook a qualitative-descriptive study [20] aimed at eliciting the requirements that our target audiences had. In particular we adopted the user-centred design principle of involving end-users in the design process and iterating based on their feedback [21]. We started with semi-structured interviews to elicit major themes, and followed this up with online testing in a larger sample to verify our findings and elicit further recommendations.

## Semi-structured interviews: methods

This work was approved by the University of Cambridge Psychology Research Ethics Committee. In August 2017, semi-structured interviews lasting ~90 min were conducted with a convenience sample (*n* = 9, 7 female, mean age 41, *SD* = 12). Participants had varying relationships to genetic testing. Four had experience as a patient, and five as health professionals. Two participants additionally had experience as patient advocates. Participants were interviewed about their experience with genetic testing results and then asked to comment on existing examples of genetic reports.

Example reports were collected to illustrate a wide variety of approaches, including some from direct-to-consumer genetic testing. Examples came from existing genetic test reports and guidelines in current usage in the UK, US and Europe to assess the range of different styles of design, wording and length employed. These included ACGS, ACMG, and ESHG guidelines and sample reports [3, 22], as well as suggested reports in the literature from Scheuner et al. [23], Dorschner et al. [15], Williams et al. [11], and report examples from GeneDX, 23andme, Partners Healthcare, and Lineagen.

The interviews were divided into two phases. Initially we asked about participants’ experience with genetic reports in their personal and professional capacities. In the second phase we sought participants’ views on the example reports addressing a number of prepared topics including the overall visual impression and appropriateness of the length, the level of the language and ease of understanding, the ‘actionability’ of the information given, and what degree of trust the report engendered. In addition, we sought comments from participants on a prototype outline based on our review of the literature, which was iteratively updated based on comments made in interviews.

Interviews were conducted by a combination of one or more of three authors (HG, AF & GF) and notes were taken during the interview. Audio was recorded for all interviews. We adopted a descriptive qualitative approach [20] with elements of a thematic analysis. Any comments from the recordings that could form the basis of a recommendation for a laboratory test report were transcribed. These comments were then aggregated for all participants and an initial code applied. Codes were then summarised into themes. In applying a descriptive qualitative approach we sought to produce a record of suggestions with minimal interpretation.

## Semi-structured interviews: results

We identified thirteen key recommendations, shown in Table 1 (See Supplementary Materials for full list). Four of these were made unanimously in all the interviews (where *n* = 9).

**Table 1:** A summary of recommendations from the semi-structured interviews that were made independently by a majority of interviewees (*n* ≥ 5). A full table of all recommendations is in the Supplementary Materials.

Grouping	Recommendation	*n*	Detail
Comm. style	Make reports easier for non-specialists to understand	9	Use layman’s terms, avoid jargon, most reports are incomprehensible even to (non-specialist) medical professionals
Structure & appearance	Consider the structure and appearance of the document	9	The structure and appearance of the document affect understanding, and ease of reading
Structure & appearance	Make the result prominent	9	The result of the test should stand out and be easily found within the document
Structure & appearance	Keep technical test details separate	9	Put technical details such as test methodology into a separate section
Content	Provide an ‘actions to be taken’ section	8	Include a section of recommendations and concrete next steps
Content	Provide sources of further information and support	8	Provide sources of authoritative information, especially on the condition, communicating the result to others and obtaining support including genetic counselling and peer support
Content	Provide a ‘what this result means’ section	7	Explain what the implications of the result are (diagnosis, risks, treatment, family)
Content	Ensure the result wording is unambiguous	6	Make the result as unambiguous as possible. Use plain language
Structure & appearance	Use colour to make things clear and easy to read	6	Colours help with understanding and appearance of document
Structure & appearance	Keep reports as short and simple as possible	6	Avoid dense blocks of text and lengthy reports as much as possible
Structure & appearance	Don’t dilute the main message	5	Don’t intersperse key messages with genetics explainers or technical details
Comm. style	Provide patients with all information	5	Patients should receive all of the information resulting from the test including technical details
Structure & appearance	Present result in neutral terms	5	Don’t use ‘positive’ or ‘negative’, or colour-code results. Aim rather for a statement of fact.

The first unanimous recommendation concerns the fact that existing genetic test reports are very difficult, if not impossible, for patients and non-specialist clinicians to understand. The main action required is the use of plain language and the avoidance of jargon. The second concerns the appearance and structure of the document itself. The clarity and flow of section headings can greatly enhance the ease of reading and comprehension. Recommendation three was to make the result of the test prominent in the report. This means that the result of the test should stand out, and preferably be the first thing the eye is drawn to. The final unanimous recommendation was to separate the technical methodological details of the genetic test conducted from the main message to be communicated to non-specialist audiences.

The recommendations in Table 1 fall broadly into three groupings. The first of these is communication style which captures a desire for plain language, the use of lay terms and the avoidance of jargon where possible. Interviewees also expressed a desire for a slightly more personal tone, for example, using ‘your’ rather than ‘the proband’s’.

The content grouping addresses the key content that patients and non-specialist clinicians seek in a report. The three main sections are: ‘What the result means’, ‘What actions should be taken’, and ‘where can further information and support be found’. The first section addresses the interpretation of the test result, including its implications for diagnosis and prognosis. The second section concerns actions to be taken, such as genetic counselling, whether testing of relatives is recommended, and accessing treatment. The final section concerns the provision of sources of further information and support. These include links to information about any diagnosed conditions, information about genetic counselling, information about communicating results to family and employers, and information on support that might be available, in particular peer support from other people similarly affected. Providing trusted links to further information helps guide patients towards trustworthy online information; patients are otherwise likely to search for online guidance themselves, which can lead to distressing and misleading information.

The structure and appearance grouping relates to the design of the document. This is important not because it is aesthetically pleasing but because it helps readers understand the content of the document. Clear section headings convey a useful hierarchy of information, documents that flow simply from top to bottom without large dense blocks of text avoid overwhelming readers. These issues are particularly important given the sometimes immensely emotive and stressful nature of obtaining and making sense of a genetic test result.

The position of technical information concerning variants and test methodology is important to consider. It was clear that this information should be contained in a patient-facing report, but that it should be clearly separated in order to allow patients and non-specialists to extract the key content they are looking for quickly and unambiguously.

Many patients also reiterated that even a well-designed report should certainly not take the place of a face-to-face meeting in which the results are communicated by a healthcare professional.

## Online survey: methods

We generated two prototype designs (see Supplementary Materials) incorporating the unanimous recommendations from the interviews, but differing in the tone and the amount of text they contained, and the presence or absence of a graphic to convey risk. The difference between the reports was designed to elicit comments on the extent that graphic design features were desirable and acceptable. The reports contained a pathogenic result for a fictitious condition ‘Brendt Syndrome’ causing an increased risk in bowel cancer. Page 1 of the report addressed the issues raised in the interviews, and page 2 contained technical and methodological details of the test.

With these prototype templates in place we sought further feedback by asking participants to complete an online survey rating different aspects of the reports. We recruited 28 patients (26 female, mean age 41, *SD* = 8), 29 non-specialist clinicians (25 female, mean age 43, *SD* = 12) and 49 members of the general public (35 female, mean age 33, *SD* *=* 10) to view our prototype reports and answer questions about them online. The patient group was recruited by advertising via support groups and advocacy organisations. Eighty-nine percent of these participants reported that they or a family member had received a genetic test report. The general public were recruited from prolific.ac, an online participant pool. Twenty percent of these participants reported having received the results of a genetic test. In the clinician group, 86% reported some professional experience of receiving genetic test reports.

Participants rated the following statements on a seven-point Likert scale: ‘I understand the results’; ‘I understand the actions [the patient/I] could take’; ‘I understand how the risk of developing cancer has changed’; ‘I would trust the results are correct’; ‘The language used is appropriate’; ‘The appearance of the report (colours, design) feels appropriate’; ‘I would want to see the technical information on Page 2’.

Free text fields in the survey also allowed participants to comment on the forms under the following categories: Questions you would still have; Trust in the result; Appearance and structure of the report; Ease of finding information; and Ease of comprehension. As with the interviews, these data were analysed using a qualitative description approach [20] with an abbreviated thematic analysis. We first coded each comment and then classified these into themes.

## Online survey: results

**Fig. 1:**
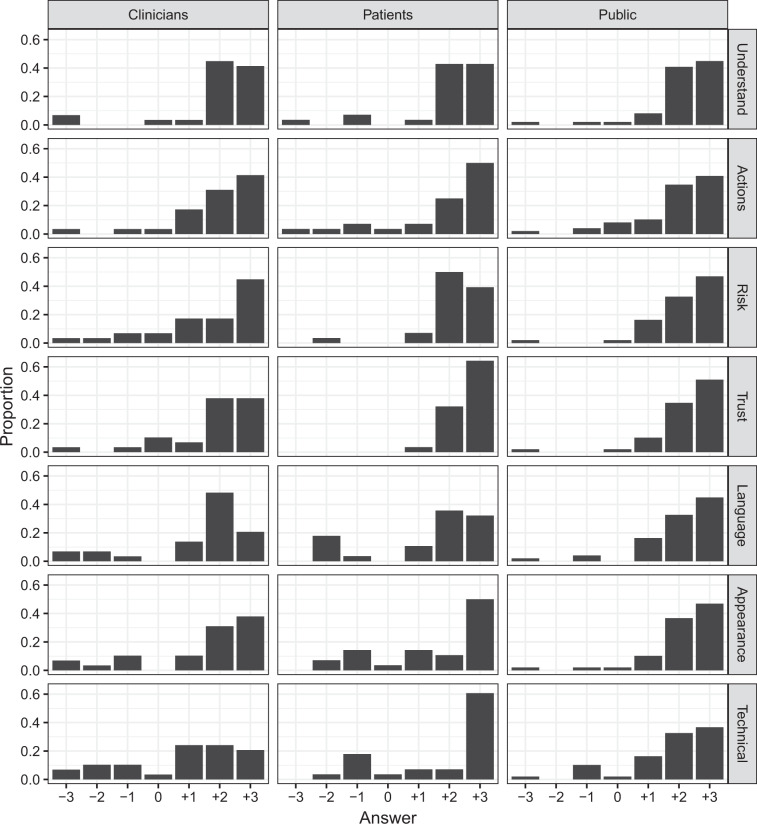
Participants’ ratings of the ‘graphic’ prototype. The *x* axis spans −3 (Completely disagree) to +3 (Completely agree) via 0 (Neither agree nor disagree). Ratings for the other prototype follow the same pattern and are included in the Supplementary Materials.

Ratings for the prototype with a risk depiction and more graphic design features, were broadly similar to those for the prototype with more text. For both, the large majority of participants rated the reports favourably. This was true across all of the features we probed, and was broadly similar for patients, clinicians, and the general public. Participants’ Likert ratings of the graphic prototype are shown in Fig. 1. The same pattern in ratings was observed for the text prototype (see Supplementary Materials).

Table 2 lists 26 themes summarising a total of 478 initially coded comments. There were four broad groupings of the themes. For the *content* themes, respondents mainly wanted more information and detail on the topics that were already present in the report. In particular, they wanted more information on actionable next steps they could take. Respondents also wanted more information about the syndrome itself. Respondents were in favour of using graphics to explain and contextualise risk. Finally, respondents were sensitive to ambiguity in the phrasing of the result, leading to comments like ‘do I have it or not?’ and questions around whether retesting would be needed.

**Table 2:** Summary of themes derived from online comments and the number of endorsements from each participant group.

Group	Theme	Clinicians (*n* = 29)	Patients (*n* = 28)	Public (*n* = 49)
Content	Would want more information about topics addressed in report (family implications, screening, treatment, next steps)	20	17	39
Content	The use of simple diagrams and figures is helpful. Avoid unfamiliar designs	11	13	15
Content	Want more detailed information about syndrome/condition (e.g. prognosis, prevalence)	5	6	9
Content	Helpful to include separated technical section	5	5	4
Content	Ambiguous wording of result unhelpful	4	0	2
Content	Would like more information about the test statistics (sensitivity, specificity etc.)	4	1	1
Content	Technical section may cause fear or confusion	3	1	0
Content	Include glossary to help with technical terms	0	2	0
Content	Unhelpful to include technical section	2	0	0
Content	Inclusion of patient details useful	0	1	0
Design	Clearly labelled sections, white space, avoiding columns, and avoiding dense blocks of text, all help with comprehension	24	22	45
Design	The appropriate use of colour helps delineate sections, but avoid it in communicating test result	12	12	26
Design	Prominence of result helpful	2	4	2
Design	Prominence of result alarming/stark	2	0	3
Design	Use large enough font	1	0	1
Comm. Style	Be concise and clear, with a personal tone, but avoid brevity at the expense of important detail.	14	14	10
Comm. Style	Patient section easy to understand, technical section is difficult	11	6	7
Comm. Style	Technical details difficult to understand	6	5	12
Comm. Style	Use lay language and avoid jargon	4	6	4
Comm. Style	Make clear who the audience is for the technical section	2	0	0
Trust	Trust of result based on technical aspects of test	7	4	5
Trust	Trust result because sourced by NHS	4	0	12
Trust	Trust of result based on appearance of document	6	0	4
Trust	Trust if confirmed by another test	1	1	2
Trust	Trust result because signed	1	0	2
Trust	Trust based on comprehension of report	0	0	2

On the design group of themes, the overwhelming response was that people appreciated a document that was simple to look at and navigate. This means a document that flows from top to bottom without columns or text boxes. It also means clearly delineating sections and avoiding dense blocks of text. The use of colour to differentiate sections of the document was appreciated, but less so in the communication of the result. Respondents from all three groups identified the grey box containing the result in one of the reports as ‘foreboding’ and ‘sinister’.

The grouping of communication style themes reflected a desire for lay terms and avoiding jargon. Many respondents identified that the first page was easy to understand and the second (technical page) was very difficult to understand. Many respondents also identified that despite this they would still like the technical details included in the report. Of the two reports people preferred the tone of the ‘text’ version as it was felt to be more personal and less ‘brusque’.

On the final grouping of trust, respondents commented on the professional appearance of the document and the fact that it was sourced by the UK’s National Health Service (NHS). These comments reflect the fact that appearing authoritative inspires trust. By contrast, some participants determined their trust in the results by assessing the statistical claims in the document. For example, respondents identified the test sensitivity (99%) as a reason to trust the result. Others found reason not to trust the result because the technical section stated the variant had not previously been found in that laboratory.

## Overall recommendations

Table 3 shows a summary of recommendations for the high-level content and structure of genetic reports suitable for patients and non-specialists that came out of our work. We emphasise that such reports are not designed to replace a face-to-face consultation between a patient and a healthcare professional. These reports may improve the experience of consultations and should be given to a patient as a take-home, not sent before a consultation.

**Table 3:** The main recommendations on the design of genetic reports that came out of this study.

Recommendation	Detail
Use lay language wherever possible	Avoid technical terms and words that can be interpreted differently by people with different backgrounds or expectations (such as ‘positive’ and ‘negative’). Don’t let brevity lead to ambiguity (e.g. ‘consistent with’) – test your wording with a lay audience to see what they would understand by it.
Employ simple design considerations such as white space, colour and clearly labelled sections	Good design can enhance trust, ease of comprehension, and lead to reduction in stress. Single column text was preferred for ease of reading. Avoid embellishments, except important logos to show the provenance of results.
Use the layout: Result > What it means > Actions > More support > Technical info.	The order of information in the report is important, and this layout was universally liked. Providing details of where to find more information and support from others is important to help steer patients through the online world, and to give them social support. Support groups should be accredited if included in a report.
The appropriate use of graphics is helpful	Graphics can help people understand numbers, or put risks into context.
Unambiguous result	Make the wording of the result as unambiguous as possible, or if the result is inherently ambiguous, explain the source of ambiguity.
Use a neutral and factual presentation of the result	Don’t use colour-coding or language to indicate whether a result is ‘good news’ or ‘bad news’ – that interpretation could be different for different people.
Use a personal tone in communication style	Address the report to the patient, using the second person (‘your…’) not the third person (‘the patient’s…’). Clinicians don’t mind reading this style, and it makes it better for the patient.
Separate, but include, the technical methodological details of the test	Patients want to know the technical details at least as much as clinicians, and including them makes the report useful for the patient’s records - particularly if they move to a new healthcare provider – and for treatment plans. But clearly label sections that are not necessary for the patient to understand so that they don’t worry that they don’t.
Communicate absolute pre- and post- risks with population for comparison. Include both framings.	If a test increases or decreases the chance of something, it is important to put those risk changes in absolute terms and in the context of the general population. What is the chance of someone in the general population compared with someone with this known genetic make-up? Frame it both positively and negatively (how many people will this happen to, and how many won’t it happen to, out of 100?)
Don’t include information that is unnecessary to understanding the key message	Although it’s tempting to attempt explain inheritance or genetics to give background to the information being provided, that is unnecessary for people to understand the result. Leave that to face-to-face genetic counselling and additional patient information leaflets.
Include the patient’s details and the context of the test on each page	Ensure the patient’s details, indication for testing, circulation and contacts of the laboratory are at the top of every page to allow instant checking that this is the ‘right report’ and who a patient can contact. The indication for testing will also help give context for interpretation for both patient and clinician.

**Fig. 2:**
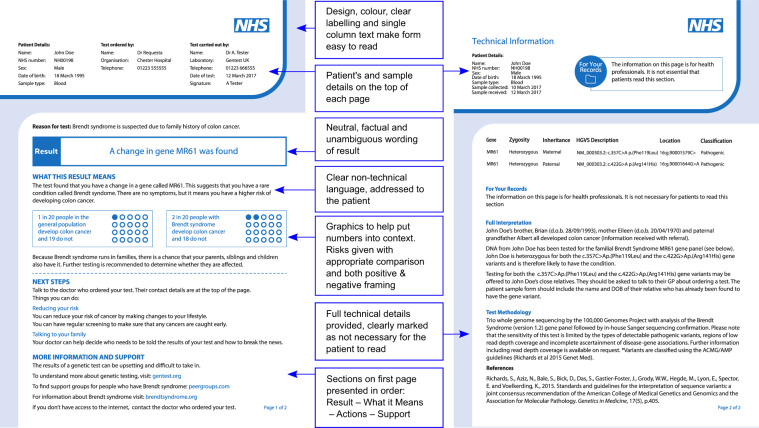
An illustration of the recommendations in action. N.B. The NHS logo does not imply endorsement or funding of this work by the National Health Service.

Figure 2 shows an example report applying some of the recommendations. Much of the more detailed information that participants wanted from reports pertains to condition-, test- or result-specific scenarios. We therefore, as an over-arching recommendation, suggest that the following should be seen as a solid foundation upon which further consultation with stakeholders can help identify the additional detail that would be necessary (e.g. identifying appropriate peer support groups, or testing how results should be worded for very different types of condition such as autism, Huntington’s or raised cancer risks). When providing information about support groups, it will be important that these have passed some form of quality check. One potential standard in the UK is Quality Mark currently being piloted by the Patient Information Forum, and which aims to help patients identify information they can trust.

These recommendations are in principle compliant with ISO15189 [24]. However, report providers should check that any final report they develop is compliant with the appropriate standards and regulations.

## Discussion

There is clearly a need for an empirically-tested template for genetic or genomic results that communicates equally well to specialist and non-specialist clinicians and patients. A review of the literature and existing reports suggested that none currently existed, and that there were clear gaps in our knowledge of what information, language and graphical design was required to construct this.

We have developed a set of recommendations and an example template that contains the major elements most important to the patients, clinicians and specialists who receive reports. There was generally broad consensus within each group as to what was important to them, and we believe the recommendations can be implemented without compromising the information required by each audience. It should be noted that there was a bias toward female volunteers in our sample, and although males did take part at all stages, any future work implementing these recommendations would benefit from a more equal balance.

Our recommendations echo many findings from the existing literature. In particular, we have created a ‘patient friendly report’ [8] which has shown with appropriate language that non-specialists perceive that they understand results and know which actions they can take, addressing concerns in Stuckey et al. [10], Williams et al. [11], and Joseph et al. [9]. Risk was effectively communicated by implementing recommended practice (raised in Lautenbach et al. [13]), and our report uses favourable graphical design while remaining appropriate (highlighted in Shaer et al. [14], and apparent in direct-to-consumer reports). Finally, we included necessary technical information and limitations (as raised in Dorschner et al. [15] and Kelman et al. [16]).

This work, however, concentrated on the design of the report template rather than the specific wording that it would carry. There are many different types of genetic or genomic test result—from diagnostic testing for patients with a condition for which they are seeking a genetic explanation, to incidental findings from a genomic test with no relevant family history, and even polygenic risk scores. This study also does not address whether or how to communicate more complex issues such as finding variants of unknown significance or when re-analysis of a sample leads to new findings. Each of these types of testing scenario will demand quite different wordings and explanations. For example, some will be attempting to convey a possible increased risk of an event with several layers of uncertainty (purely aleatoric uncertainty which can be portrayed as a probability; epistemic uncertainty about the strength and quality of the evidence surrounding the influence of the gene variant on future risk; uncertainty related to the sensitivity and specificity of the test etc.), each needing quite a different approach. This would be different from, say, a carrier testing result where the gene variant has very high penetrance and is well characterised so the communication is mainly about the chances of inheritance in future children.

To ascertain how best to illustrate and explain results in the many complex reporting scenarios beyond the scope of this research, it will be critical that the content and formats of reports are carefully co-designed with their target audiences. Following the design of this generic template, we continued with development of wording and further design refinements, starting with templates for carrier testing, and when formally assessed against report forms in current clinical use proved clearer and more actionable for patients (see Recchia et al.) [25]. This article provides a framework for effective communication of genetic test results. In addition, Recchia et al. [25] provides a methodology for tailoring the framework to more specific testing scenarios.

The process of iteratively engaging with recipients of reports to produce this template and recommendations, is in itself analogous to shared decision-making, a concept becoming increasingly familiar in medicine. It involves a dialogue with patients to find out what is important to them and come to a mutually-agreed solution that serves their needs for clear and accurate information.

Providing patients, and their non-specialist healthcare providers, with accurate and unambiguous test results arms them with the information that they need to be able to take part in shared decision-making. Where the results do not need any further action, these forms should be able to give clear reassurance without the need for input from genetic specialists. When the results have implications that need full discussion and decision-making, they can help ensure that the patient is referred correctly to a suitably qualified professional and give them the basic information they need to take part in shared decision-making and informed consent during that consultation.

In genetics, the unit of care is often the family rather than just the individual, it is really important therefore that genetic information be conveyed to other family members accurately and having the patient understand the information makes this much more likely to happen. Indeed a number of interviewees identified that facilitating communication with others would be a particularly desirable benefit of a well-designed report. Although it is worth noting that patients do not always wish to communicate results to family members raising difficult privacy and consent issues (see section 4 in a recent report by the Joint Committee on Genomics in Medicine [26] for a discussion).

By working across multiple audiences, and hand-in-hand with those who need to implement any reports in actual clinical practice, we have sought to develop a practical and useful product which is flexible enough to be able to carry test results of many kinds, and will lead to better-informed decision-making. We also hope to have demonstrated more generally that involving the target audience in the design of a communication is an efficient and valuable way to ensure effective communication of important medical information.

## Supplementary information

Supplementary Materials
