# Supplementary material for: Recommendations for designing genetic test reports to be understood by patients and non-specialists
Source: Eur J Hum Genet. 2020 Feb 5;28(7):885–95. doi: 10.1038/s41431-020-0579-y (PMC7316722; doi:10.1038/s41431-020-0579-y)
Supplement: Supplementary file 1 — Supplementary Materials [file 41431_2020_579_MOESM1_ESM.pdf]

Supplementary Materials

Two supplementary figures and one supplementary table. All are referenced in the manuscript.

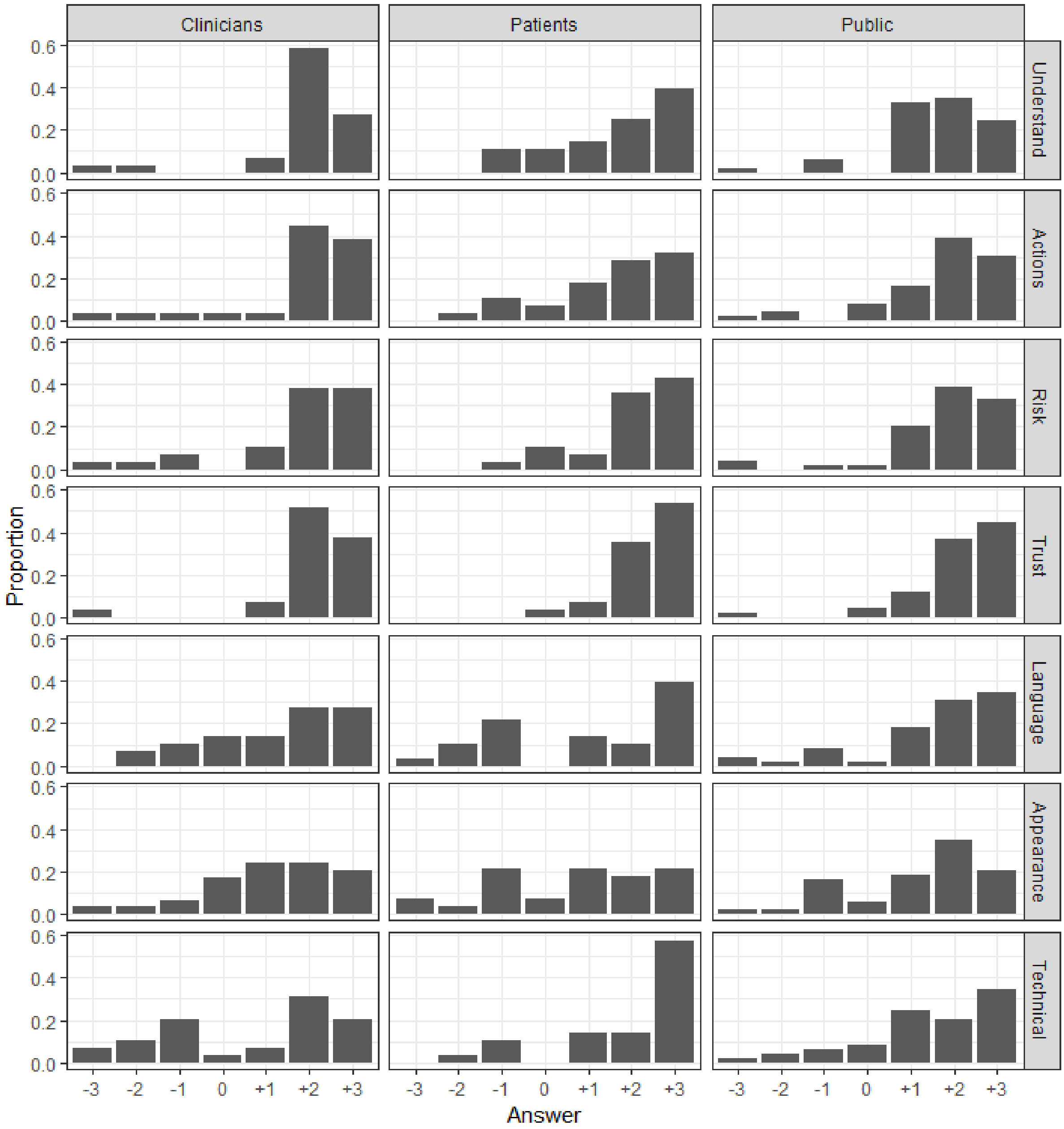

Figure S1. Results of the online feedback for the ‘text’ prototype. The x axis shows Likert scores ranging from -3 (completely disagree) through 0 (neither agree nor disagree) to +3 (completely agree)

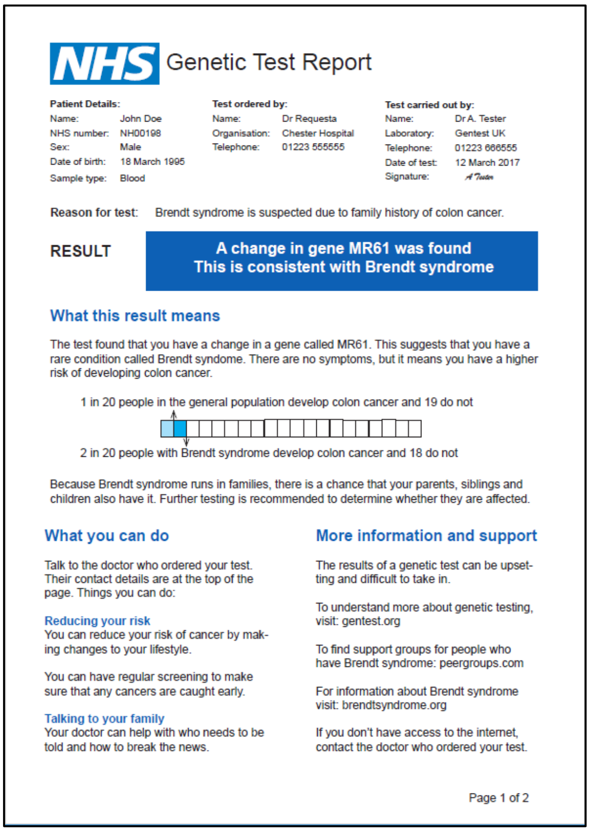

NHS Genetic Test Report

Patient Details:		Test ordered by:		Test carried out by:	
Name:	John Doe	Name:	Dr Requesta	Name:	Dr A. Tester
NHS number:	NH00198	Organisation:	Chester Hospital	Laboratory:	Gentest UK
Sex:	Male	Telephone:	01223 555555	Telephone:	01223 666555
Date of birth:	18 March 1995			Date of test:	12 March 2017
Sample type:	Blood			Signature:	A Tester

Reason for test: Brendt syndrome is suspected due to family history of colon cancer.

RESULT A change in gene MR61 was found
This is consistent with Brendt syndrome

What this result means

The test found that you have a change in a gene called MR61. This suggests that you have a rare condition called Brendt syndome. There are no symptoms, but it means you have a higher risk of developing colon cancer.

1 in 20 people in the general population develop colon cancer and 19 do not

2 in 20 people with Brendt syndrome develop colon cancer and 18 do not

Because Brendt syndrome runs in families, there is a chance that your parents, siblings and children also have it. Further testing is recommended to determine whether they are affected.

What you can do

Talk to the doctor who ordered your test. Their contact details are at the top of the page. Things you can do:

Reducing your risk
You can reduce your risk of cancer by mak-ing changes to your lifestyle.

You can have regular screening to make sure that any cancers are caught early.

Talking to your family
Your doctor can help with who needs to be told and how to break the news.

More information and support

The results of a genetic test can be upset-ting and difficult to take in.

To understand more about genetic testing, visit: gentest.org

To find support groups for people who have Brendt syndrome: peergroups.com

For information about Brendt syndrome visit: brendtsyndrome.org

If you don't have access to the internet, contact the doctor who ordered your test.

Page 1 of 2

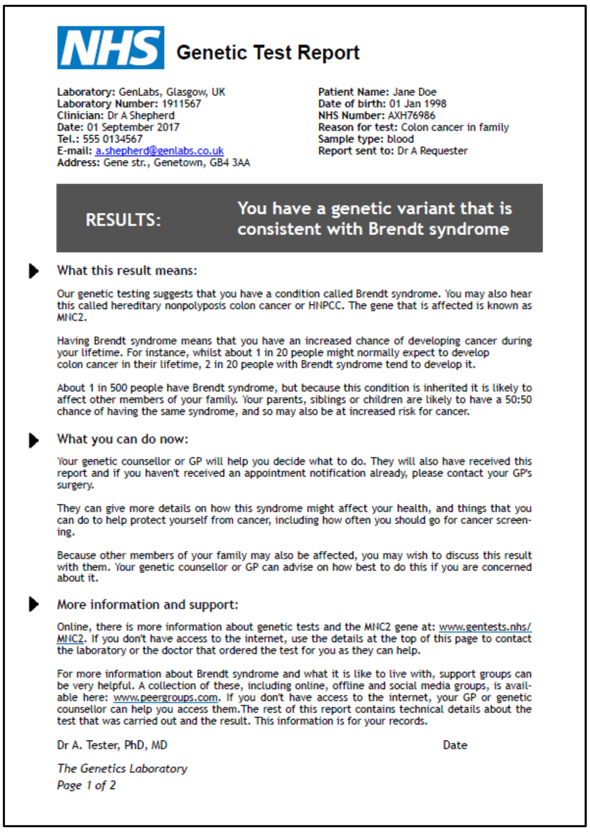

NHS Genetic Test Report

Laboratory: GenLabs, Glasgow, UK
Laboratory Number: 1911567
Clinician: Dr A Shepherd
Date: 01 September 2017
Tel.: 555 0134567
E-mail: a.shepherd@genlabs.co.uk
Address: Gene str., Genetown, GB4 3AA

Patient Name: Jane Doe
Date of birth: 01 Jan 1998
NHS Number: AXH76986
Reason for test: Colon cancer in family
Sample type: blood
Report sent to: Dr A Requester

RESULTS: You have a genetic variant that is consistent with Brendt syndrome

What this result means:

Our genetic testing suggests that you have a condition called Brendt syndrome. You may also hear this called hereditary nonpolyposis colon cancer or HNPCC. The gene that is affected is known as MNC2.

Having Brendt syndrome means that you have an increased chance of developing cancer during your lifetime. For instance, whilst about 1 in 20 people might normally expect to develop colon cancer in their lifetime, 2 in 20 people with Brendt syndrome tend to develop it.

About 1 in 500 people have Brendt syndrome, but because this condition is inherited it is likely to affect other members of your family. Your parents, siblings or children are likely to have a 50:50 chance of having the same syndrome, and so may also be at increased risk for cancer.

What you can do now:

Your genetic counsellor or GP will help you decide what to do. They will also have received this report and if you haven't received an appointment notification already, please contact your GP's surgery.

They can give more details on how this syndrome might affect your health, and things that you can do to help protect yourself from cancer, including how often you should go for cancer screen-ing.

Because other members of your family may also be affected, you may wish to discuss this result with them. Your genetic counsellor or GP can advise on how best to do this if you are concerned about it.

More information and support:

Online, there is more information about genetic tests and the MNC2 gene at: www.gentests.nhs/MNC2. If you don't have access to the internet, use the details at the top of this page to contact the laboratory or the doctor that ordered the test for you as they can help.

For more information about Brendt syndrome and what it is like to live with, support groups can be very helpful. A collection of these, including online, offline and social media groups, is avail-able here: www.peergroups.com. If you don't have access to the internet, your GP or genetic counsellor can help you access them.The rest of this report contains technical details about the test that was carried out and the result. This information is for your records.

Dr A. Tester, PhD, MD Date

The Genetics Laboratory
Page 1 of 2

Figure S2. The two prototype reports used to gather online feedback. The 'graphic' version (left) differs in that it contains less text, contains a graphic representation of risk and has more white space with more clearly separated sections. The reverse side of both forms contain technical details pertaining to the test methodology

Table S1. Full Table of recommendations from interviews with number of interviewees (n=9) endorsing each.

Grouping	Recommendation	n	Detail
Comm. Style	Make reports easier for non-specialists to understand	9	Use layman's terms, avoid jargon, most reports are incomprehensible even to (non-specialist) medical professionals
Structure & Appearance	Consider the structure and appearance of the document	9	The structure and appearance of the document affect understanding, and ease of reading
Structure & Appearance	Make the result prominent	9	The result of the test should stand out and be easily found within the document
Structure & Appearance	Keep technical test details separate	9	Put technical details such as test methodology into a separate section
Content	Provide an ‘actions to be taken’ section	8	Include a section of recommendations and concrete next steps
Content	Provide sources of further information and support	8	Provide sources of authoritative information, especially on the condition, communicating the result to others and obtaining support including genetic counselling and peer support
Content	Provide a ‘what this result means’ section	7	Explain what the implications of the result are (diagnosis, risks, treatment, family)
Content	Ensure the result wording is unambiguous	6	Make the result as unambiguous as possible. Use plain language
Structure & Appearance	Use colour to make things clear and easy to read	6	Colours help with understanding and appearance of document
Structure & Appearance	Keep reports as short and simple as possible	6	Avoid dense blocks of text and lengthy reports as much as possible
Structure & Appearance	Don’t dilute the main message	5	Don't intersperse key messages with genetics explainers or technical details
Comm. Style	Provide patients with all information	5	Patients should receive all of the information resulting from the test including technical details
Structure & Appearance	Present result in neutral terms	5	Don't use 'positive' or 'negative' or colour-code results. Aim rather for a statement of fact.
Comm. Style	Contextualise risks	4	Provide population baseline risks, and use absolute risks

Structure & Appearance	Indicate document hierarchy	4	The document should have a clear hierarchy of information so that patient is clear about what needs to be understood, and what does not.
Comm. Style	More personal	4	Make the document less clinical, e.g., use 'your' instead of 'patient' or 'proband'
Content	Show patient details	4	Have the patient's details visible on the form (allows patient to be sure is their result)
Content	Time course	4	Include time frames for implications of the result and for next steps to be taken by the patient
Comm. Style	Balanced tone	3	Document needs to strike a balance between serious and personal
Content	Include risks	3	Include information about elevated risks resulting from a variant
Comm. Style	Multiple formats	3	Explain risks, and other quantitative information, in multiple formats so that as many people as possible will understand
Structure & Appearance	No document adornments	3	Avoid side-bars, boxes or call-outs
Comm. Style	Patient first	3	If the report has patient and clinician sections, then put patient first
Content	Family implications	2	A 'what this means' for relatives section
Content	Future changes	2	Be clear that as science advances results can be reinterpreted
Content	Include signature	2	Doctor or scientist’s signature provides reassurance
Structure & Appearance	Order as wanted	2	Order the document sections in the order the patient will want the info
Comm. Style	Avoid terms with colloquial meaning	2	Terms like 'mutant' can feel stigmatising.
Content	Use visuals	2	Graphics and other visuals are good for explaining technical issues
Content	Why test ordered	2	Include test indication, but avoid jargon.
Content	Contact details	1	Provide contact details so patient can contact laboratory, or clinician that ordered test
Content	Provide diagnosis	1	State confirmation of diagnosis where appropriate
Structure & Appearance	Result first	1	Result of test should appear first in document
Content	Technical limitations	1	Include technical limitations of test

Comm. Style	Text size	1	Make font large enough to be read
Structure & Appearance	Use document adornments	1	Do use columns and text boxes etc. But see above (more interviewees disagree)
Structure & Appearance	Use logo	1	Laboratory or health authority logo adds air of professionalism and increases trust
Content	Volunteer opportunities	1	Link to opportunities to take part in research on conditions associated with variant
Comm. Style	Provide access to information offline	1	Be aware some patients do not have access to internet
